# The Role of Aquaporins in pH-Dependent Germination of *Rhizopus delemar* Spores

**DOI:** 10.1371/journal.pone.0150543

**Published:** 2016-03-09

**Authors:** Tidhar Turgeman, Arava Shatil-Cohen, Menachem Moshelion, Paula Teper-Bamnolker, Christopher D. Skory, Amnon Lichter, Dani Eshel

**Affiliations:** 1 Department of Postharvest Sciences of Fresh Produce, Agricultural Research Organization (ARO), The Volcani Center, Bet-Dagan, Israel; 2 Department of Plant Sciences and Genetics in Agriculture, The Robert H. Smith Faculty of Agriculture, Food and Environment, The Hebrew University of Jerusalem, Rehovot, Israel; 3 Renewable Product Technology Research Unit, NTL Center for Agricultural Utilization Research, Peoria, Illinois, United States of America; Faculty of Pharmacy, University of Lisbon, PORTUGAL

## Abstract

*Rhizopus delemar* and associated species attack a wide range of fruit and vegetables after harvest. Host nutrients and acidic pH are required for optimal germination of *R*. *delemar*, and we studied how this process is triggered. Glucose induced spore swelling in an acidic environment, expressed by an up to 3-fold increase in spore diameter, whereas spore diameter was smaller in a neutral environment. When suspended in an acidic environment, the spores started to float, indicating a change in their density. Treatment of the spores with HgCl_2_, an aquaporin blocker, prevented floating and inhibited spore swelling and germ-tube emergence, indicating the importance of water uptake at the early stages of germination. Two putative candidate aquaporin-encoding genes—*RdAQP1* and *RdAQP2*—were identified in the *R*. *delemar* genome. Both presented the conserved NPA motif and six-transmembrane domain topology. Expressing *RdAQP1* and *RdAQP2* in *Arabidopsis* protoplasts increased the cells' osmotic water permeability coefficient (*P*_f_) compared to controls, indicating their role as water channels. A decrease in *R*. *delemar* aquaporin activity with increasing external pH suggested pH regulation of these proteins. Substitution of two histidine (His) residues, positioned on two loops facing the outer side of the cell, with alanine eliminated the pH sensing resulting in similar *P*_f_ values under acidic and basic conditions. Since hydration is critical for spore switching from the resting to activate state, we suggest that pH regulation of the aquaporins can regulate the initial phase of *R*. *delemar* spore germination, followed by germ-tube elongation and host-tissue infection.

## Introduction

Fungal diseases are one of the major causes of fruit and vegetable losses, with *Rhizopus* spp. having a major impact [1, 2]. *Rhizopus* infection occurs before and after harvest, and one of its characteristic features is the rapid establishment of infection on diverse hosts with a preference for fleshy fruit and vegetables. The large amount of fungal spores which are produced during the infection increase the probability of infecting wounds in subsequent disease cycles and the resting state ensures the preservation of fungal viability under the action of unfavorable factors (reviewed by [3]). The first developmental stage in the life cycle of all filamentous fungi and in host–pathogen interactions of pathogenic fungi initiates with germination of the resting spore in response to suitable environmental conditions [4, 5]. The factors triggering spore germination are diverse; they are based on the adaptations and requirements of the fungal species and they differ among species. Wound-invading fungi such as *Rhizopus delemar* and *Penicillium expansum* require nutrients that often originate from host wounds to induce spore germination [6, 7]. Fungal spores have been optimally induced for germination in the presence of a mixture of amino acids, as in the case of *Rhizopus oligosporus* [8] and *Aspergillus flavus* [9]. Alternatively, single amino acids can act as stimulators of germination, as found, for example, for *Trichophyton mentagrophytes* microconidia [10–12]. Mixtures of nitrogen and carbon sources have been found to enhance germination in several pathogenic fungi: glucose combined with ammonium chloride (NH_4_Cl) induced a high percentage of *A*. *flavus* conidial germination [9]. Optimal germination of *Rhizopus arrhizus* spores has been observed in the presence of carbon and nitrogen sources together with phosphate, sulfate, potassium and magnesium ions [13]. Hydration, attachment to hydrophobic surfaces, simple sugars and minerals can be additional stimulators for spore germination [14, 15].

Apart from nutrient availability, spore germination is critically influenced by environmental conditions, e.g., temperature, relative humidity and water activity [16–20]. Fungal ability to sense and respond to the environment is critical for their survival. For species with a tight association to host organisms (pathogens, symbionts or commensals), adaptation and response to the host's microenvironment are particularly important. One key environmental factor to which fungi must respond is ambient pH. Changes in the surrounding pH can potentially affect cellular mechanisms at both the transcriptional and functional levels (e.g. by altering micronutrient availability, protein function or membrane potential) and can thus determine the fate of microorganisms [21, 22]. Spores of various fungi are known to germinate in a specific pH range [8, 23–27]. For several pathogenic fungi, such as *Penicillium*, *Aspergillus* and *Rhizopus* spp., the optimal pH values for germination are acidic, ranging between 4.0 and 6.0 [25, 27].

Previous studies have linked the inhibition of fungal growth when they are outside the optimal pH range to changes in the efficiency of enzyme activities, nutrient availability and the proton gradient across the plasma membrane [28, 29]. Spore swelling at the primary stage of germination involves hydration of the spore by water uptake from the environment, as reported for *Rhizopus*, *Piptocephalis* and *Zygorhynchus* [13, 30]. The constant rate of water uptake per unit of spore surface area as the volume of the spore increases led researchers to the assumption that water uptake during this stage is a regulated process which does not depend on diffusion (reviewed by [31]). Further support for this assumption came from the spores of powdery mildews, which can take up water rapidly [32] but lose water slowly, even over desiccants [33]. In addition, metabolic inhibitors such as sodium azide have been found to prevent the swelling, indicating a metabolic role in the water-absorption and swelling process [5]. As an important developmental stage during the life cycle of fungi, spore germination has been widely investigated. Nevertheless, the mechanism by which spores regulate water uptake during the early stages of germination has not yet been established in fungi. Our objective was to study the role of aquaporins in the early events leading to spore germination, and the effect of external pH on their function.

## Materials and Methods

### Fungal strains and growth conditions

The *Rhizopus delemar* strain used in this study was 99–880 from the collection of Dr. Skory. Fungal cultures were routinely grown at 25°C on potato dextrose agar (PDA) plates. PDA was prepared by dissolving 39 g/l of PDA powder (Difco Laboratories, Detroit, MI) and 25 mg/l of chloramphenicol (Sigma, Rehovot, Israel) in purified water, sterilizing it with an autoclave and pouring the medium into 90-mm Petri dishes. Spores were harvested from 1- to 2-week-old PDA plates by gently rubbing the mycelia with a Drigalski spatula and sterile water. The suspension was filtered through eight layers of gauze cloth and spore concentration was determined by counting with a hemacytometer and light microscope. The spore concentration was adjusted to the desired value by adding sterile water.

### Identification of germination inducers in purified sweet potato extract

Preparation, extraction and purification of sweet potato (*Ipomoea batatas* L. cv. Georgia Jet) active fraction (SPAF) were performed according to Turgeman et al. [7]. Fungal spores were treated with SPAF in purified water as the test treatment or purified water as a negative control. To examine the effect of pH on *R*. *delemar* spore germination, 20 mg/ml SPAF solution was adjusted for pH with 0.1 M HCl or 1 M NaOH and the level of osmolarity was 40+/-2 mOsm for pH 2.5–8.5 (using a Vapro 5600 vapor pressure osmometer, Wescor, Logan, UT). Spore solution (0.5 ml) at a final concentration of 5 x 10^5^ spore/ml was transferred into 50-ml tubes containing 0.5 ml SPAF and 4 ml purified water, or 4.5 ml purified water. The treated spores were then incubated in an orbital shaker at 30°C and 100 rpm. Spore germination was examined after 3 h and 24 h by mixing the solution and placing a 10-μl drop (in duplicate) onto a glass slide (Diagnostic microscope slides, Marienfeld-Superior, Lauda, ​​Germany) and observing it under a light microscope (Eclipse 50i, Nikon, Japan). For photography, 1 ml of the sample at each studied time point was spun down (4°C, 2500 x *g*) and the spores were fixed with 3.7% (v/v) formaldehyde to prevent further growth. Pictures were taken at the same magnification (X100) with a digital camera (DS-Fi1 Nikon, Japan) mounted on the microscope.

### HgCl_2_ treatment

To determine whether aquaporin (AQP) proteins are involved in water uptake during the early stage of germination, spores were incubated for 5 min with 40 μM of the AQP blocker HgCl_2_ (Sigma-Aldrich), followed by two washes in double-distilled water and suspension in 20 mg/ml SPAF to induce germination. Spores were incubated with 5 μM of the reducing agent 2-β-mercaptoethanol (2ME) (Sigma-Aldrich) for 5 min to reverse the inhibition effect.

### RNA extraction and cDNA synthesis

Following the induction of spore germination using SPAF, *R*. *delemar* spores were harvested at seven time points, with 30-min intervals. The spores were frozen in liquid nitrogen and stored at -80°C. Total RNA was extracted from ~20 mg tissue using the SV total RNA isolation kit according to the manufacturer's instructions (Promega, Madison, WI). For cDNA synthesis, 1 μg total RNA and 0.1 μM random hexamer primers were heated for 5 min at 65°C and snap-chilled on ice. The following components were added to the reaction mixture: 0.2 mM dNTP mixture (Fermentas, Glen Burnie, MD), M-MuLV-reverse transcriptase (RT) buffer (1X final concentration), 40 U of RNase M-MuLV inhibitor (Fermentas), 200 U of M-MuLV-RT enzyme (Fermentas) and DEPC-treated water to a reaction volume of 21 μl. The reaction was incubated at 42°C for 60 min then 70°C for 10 min.

### Quantitative real-time PCR

Quantitative real-time PCR (qPCR) was performed on cDNA reverse-transcribed from the RNA extracted from *R*. *delemar* spores. The *Rhizopus 18S* ribosomal RNA served as the reference gene for RNA amounts, and was amplified using specific primers: 5′-GACGCAAGGCTGAAACTTAAAGG-3′ (F) and 5′-CCCCGTGTTGAGTCAAATTAAGC-3′ (R) [34]. For the determination of *R*. *delemar* AQP [GenBank accession numbers EIE90948.1 (*RdAQP1*) and EIE91236.1 (*RdAQP2*)] expression, the following primers were used: 5′-TCAACTGTTGGGTGCATTTGC-3′ (F) and 5′-TGACCGCCATCGAACTGAAC-3′ (R) and 5′-AACCTCTTCCCTTGGTTCAGG-3′ (F) and 5′-GGATTCAGATGACCGCCAGA-3′ (R), respectively. qPCR amplification conditions were as previously described [35]. The expression data were analyzed by the relative standard curve method using the ΔΔCT method [36]. All experiments were carried out with a non-template control and repeated three times, each with four technical repeats.

### Phylogenetic analysis

231 fungal major intrinsic proteins (MIPs) from 88 fungal species representing four phyla (Basidiomicota, Ascomycota, Glomerumycota and Zygomycota) (reviewed in [37]) were phylogenetically analyzed and a phylogenetic tree was constructed. Deduced amino acid sequences were aligned using ClustalW, followed by analysis using neighbor-joining in MEGA 4.0.

#### Expression plasmids

The coding regions of *R*. *delemar RdAQP1* and *RdAQP2* [ATG to stop codon according to GenBank accession numbers EIE90948.1 (918 bp) and EIE91236.1 (951 bp), respectively] were chemically synthesized (Genewiz, Plainfield, NJ). In addition, two His residues of the gene *RdAQP1* in positions 85 and 275 (H85 and H275, respectively) were replaced with alanine (A) and synthesized as well. All fragments were cloned separately into plasmid pDONR221 using the standard BP clonase2 reaction of the Gateway cloning system (Invitrogen, Carlsbad, CA). The insert of the entry clone was verified by sequencing. All three fragments were subcloned into expression vector pK7WG2D1 (35S promoter and 35S terminator; 50 μg/ml kanamycin as a selection marker) for constitutive expression in Arabidopsis protoplasts. In addition, *RdAQP1* and *RdAQP2*, fused to N-terminal GFP, were subcloned into pK7FWG2 (35S promoter and 35S terminator) for constitutive expression in *R*. *delamar* protoplasts, using standard LR clonase2 according to a Gateway Vector Conversion protocol (Invitrogen). The nucleotide sequences of all constructs were confirmed by sequencing.

### *Arabidopsis* protoplast isolation and transformation

*Arabidopsis thaliana* (Col-0) seeds were sterilized, cold-treated (4°C) and germinated in pots. Plants were grown in a 20°C to 22°C growth chamber under short-day conditions (8 h light and 16 h dark) for 1 month. For protoplast isolation, the lower leaf epidermis was peeled off at the leaf center. The peeled leaves were cut into small squares and incubated in enzyme solution [3.3% w/w of an enzyme mix containing the following enzymes: 0.55 g cellulase (Worthington, Lakewood, NJ), 0.1 g pectolyase (Karlan, Phoenix, AZ), 0.33 g polyvinylpyrrolidone K 30 (Sigma-Aldrich), 0.33 g BSA (Sigma-Aldrich)] in solution containing 10 mM KCl, 1 mM CaCl_2_, 540 mM D-sorbitol and 8 mM 2-(*N*-morpholine)-ethanesulfonic acid (MES), pH 5.7. After 20 min of incubation at 28°C, the leaf tissue was transferred to fresh isotonic solution. The remaining tissues were gently shaken to release the protoplasts into the solution and removed. The protoplasts were collected with a clipped-off 100-μl pipette tip into a 1.5-ml tube. Protoplasts were transformed with the various constructs (under a 35S promoter) using polyethyleneglycol (PEG) as described by Locatelli et al. [38]. Protoplasts transiently expressing cytosolic GFP were used as a control. Imaging of GFP was performed with a motorized epifluorescence inverted microscope (Olympus-IX8 Cell-R, Tokyo, Japan) with the following features: objective lens, plan apochromat, 60X, oil immersion, and a numerical aperture of 1.42. The CCD camera was a 12-bit Orca-AG (Hamamatsu, Hamamatsu city, Japan). The filter sets were GFP-3035B-000 and TXRED-4040B, with zero pixel shift (Semrock, Rochester, NY). All images were processed using Olympus imaging software Cell-R for Windows. For detailed description (video article) of the protoplast isolation and *P*_f_ measurement, please see Shatil-Cohen et al. [39].

### *R*. *delemar* protoplast isolation and transformation

Fungal transformation of protoplasts was performed as previously described [40]. Briefly, *R*. *delemar* spores originated from 7-day-old cultures (5 × 10^7^ spores/ml) were germinated in 20 mg/ml SPAF solution for 4 h and filtered through Whatman No. 1 filter paper. The collected spores were then transferred to a filtered (0.45-μm filter) enzyme mixture prepared in 15 ml of osmotic medium (147.9 g MgSO_4_ in 10 mM NaPO_4_ buffer adjusted to pH 5.8 in 500 ml) containing 0.1 g lysing enzyme from *Trichoderma harzianum* (Sigma-Aldrich), 0.05 g cellulase from *Aspergillus niger* (Fluka Japan, Tokyo, Japan), 0.1 g novozyme (InterSpex Products, Inc., Foster City, CA), and 0.1 g Yatalase (Takara, Shiga, Japan). The spores were shaken overnight at 27°C and 50 rpm and transferred to osmotic buffer that enabled protoplast collection. For PEG-mediated transformation, 1–9 x 10^6^ protoplasts in 100 μl STC (1.2 M sorbitol, 10 mM Tris–HCl pH 7.5) were mixed with 10 μg of the constructs and incubated for 20 min at room temperature. Three 400-ml aliquots of 60% PEG 4000 (Sigma) containing 10 mM CaCl_2_ and 10 mM Tris–HCl (pH 7.5) were gently mixed with the protoplasts and incubated at room temperature for 20 min. The protoplasts were then pelleted at 800 x *g* for 5 min, washed with 2 ml of STC, collected by centrifugation at 800 x *g* for 5 min and resuspended in 225 ml of 20 mg/ml SPAF solution. The cell suspension was plated on PDA with 0.3% (w/v) yeast extract containing 0.6 M sucrose. After 7 days, spores were harvested and screened for GFP fluorescence using an epifluorescence inverted microscope.

### Osmotic water permeability coefficient (P_f_) measurements

To identify the GFP-labeled protoplasts, we screened the protoplast population using the above filter sets. The Pf was measured in single protoplasts from the initial (recorded) rate of their volume increase in response to hypo-osmotic challenge (a 0.25 MPa change from 600 mOsm isotonic bath solution to 500 mOsm hypotonic solution). Isotonic (600 mOsm) and hypotonic (500 mOsm) solutions containing 10 mM KCl,1 mM CaCl_2_, and 8 M 2-(N-morpholine)-ethanesulphonic acid (MES), pH 5.7 and osmolarity was adjusted with the appropriate amounts of D-sorbitol: 540 mM for the isotonic and 440 mM for the hypotonic solution. pH was adjusted using 0.1M HCl or NaOH with no detected change in osmolarity. Osmolarity verification of the solution was done within 3% of the target value using a vapor pressure osmometer (Wescor).

*P*_f_ was determined using a numerical approach, in an offline curve-fitting procedure of the PfFit program, as described previously [39, 41–44] and detailed in Moshelion et. al. [42]. Briefly, the instantaneous, initial value of the osmotic permeability of the membrane, P_fi_, was determined from the *initial* rate of volume increase, i.e., from the slope dV/dt of the linear phase (first few seconds) of the volume *vs*. time plot, using specific equations. This determination was based on the premise that the rate of bath solution exchange was instantaneous, such that the external concentration, C_out_, attained immediately its final value, and that the internal concentration, C_in_, remained at its initial value.

### In-silico topology and homology model

*In-silico* prediction of RdAQP1 and RdAQP2 topology was done using the TMHMM method (http://www.cbs.dtu.dk/services/TMHMM). A hypothetical three-dimensional structure of the RdAQP1, was based on the crystal structure of the *Saccharomyces cerevisiae* AQY1. The Swiss-Model server was used to create the model [45].

## Results

### Glucose induces *R*. *delemar* spore swelling under acidic conditions

**Fig 1:**
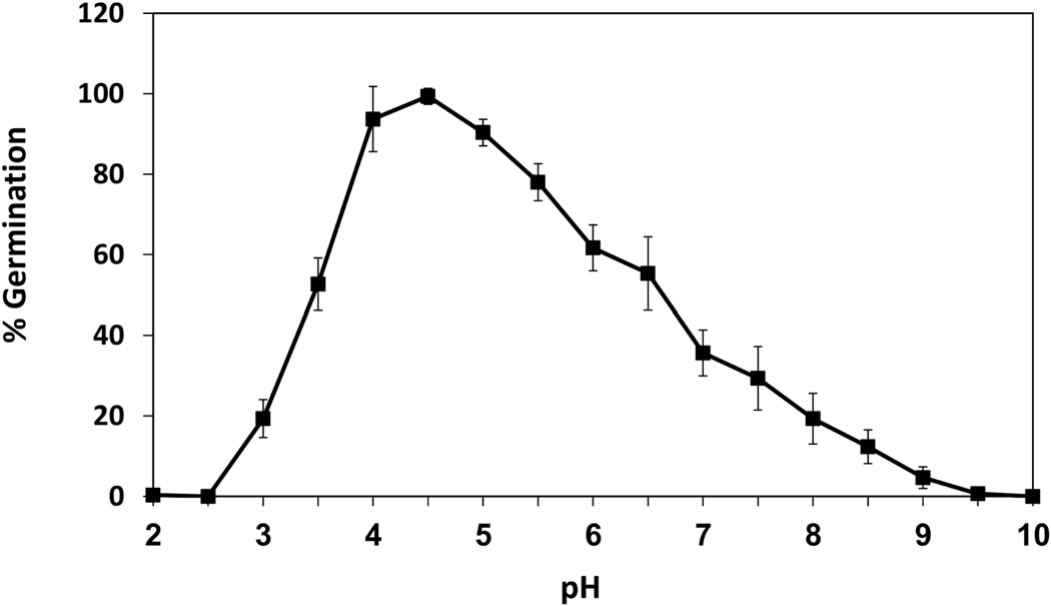
Effect of pH on *Rhizopus delemar* spore germination. Percentage of *R*. *delemar* spores germinated under different pH conditions. Spores were incubated in SPAF solution (20 mg/ml, 42°C) and scored at 6 h. Values are means ± SE (n = 500).

In a previous work, we isolated a fraction within the sweet potato extract (SPAF) that was found to induce *R*. *delemar* spore germination [7]. The pH of the SPAF was acidic (pH 4.7) and when it was modified, the optimal pH range for *R*. *delemar* spore germination was 4.0 to 5.0, with maximal germination at pH 4.5 (Fig 1). Germination was reduced to less than 20% at pH values above 8.0 and below 3.0.

In our previous study, we identified sucrose, glucose, fructose, organic acids and amino acids as the major constituents of SPAF [7]. Incubation of *R*. *delemar* spores with the separated major HPLC fractions of SPAF did not result in germination (Table 1). The pH of the fractions containing the sugar peaks (sucrose, glucose and fructose) was neutral (~7.0), whereas the fractions containing the organic acid and amino acid peaks had a pH of 4.7. As acidic pH was deemed essential for germination, the sugar fraction was acidified to pH 4.7 and incubated with *R*. *delemar* spores. The acidified glucose fraction induced spore swelling, without germ-tube emergence (Table 1), whereas acidification of the sucrose or fructose fractions did not have any effect on spore swelling or germination. Only incubation of *R*. *delemar* spores with the glucose fraction combined with amino acid and organic acid fractions enabled germ-tube emergence (Table 1). In both cases, i.e. SPAF or combined peak treatments, acidic pH caused spore swelling and a higher percentage of germ-tube emergence.

**Table 1:** Effect of major peaks resulting from HPLC fractionation of sweet potato active fraction (SPAF) on *Rhizopus delemar* spore swelling and germination. The pH of each HPLC fraction was modified to 4.7 or 7. The peaks contained: 1 –a mixture of organic and amino acids, 2 –sucrose, 3- glucose, 4 –fructose, or the combination of 1 and 3. SPAF and water served as controls. Values are means ± SE (n = 500).

*HPLC Fraction*		pH	Spore diameter (μm)	Germ-tube emergence (%)
No.	Content			
	Water	4.7	8.5±0.3 *Bb	0
		7.0	8.1±0.7 Bb	0
	SPAF	4.7	22.2±0.6 Aa	96.9±2.5 Aa
		7.0	15.7±2.1 Ab	44.2±3.3 Bb
1	Amino & organic acids	4.7	8.6±0.6 Bb	0
		7.0	8.1±0.2 Bb	0
2	Sucrose	4.7	7.9±0.7 Bb	0
		7.0	8.3±0.8 Bb	0
3	Glucose	4.7	19.5±1.9 Aa	0
		7.0	14.3±2.4 Ab	0
4	Fructose	4.7	8.9±0.3 Bb	0
		7.0	8.4±0.6 Bb	0
1+3	Amino & organic acids & Glucose	4.7	21.1±0.7 Aa	97.3±1.8 Aa
		7.0	14.6±1.5 Ab	35.7±5.7 Bb

* Within each pH level (4.7 or 7.0) different upper case letters are significantly different from each other (P≤ 0.05). Within each pair of pH level in the same fraction content (Water, SPAF, etc.), lowercase letters are significantly different from each other (P≤ 0.05).

### Inhibition of *R*. *delemar* spore swelling

**Fig 2:**
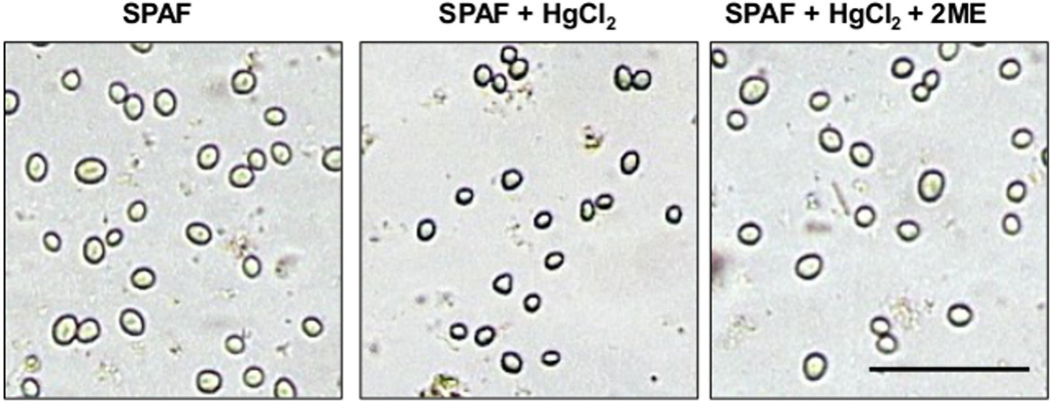
Effect of HgCl_2_, an inhibitor of AQPs function on swelling of spore of *Rhizopus delemar*. Treatment of *R*. *delemar* spores with 40 μM HgCl_2_ for 5 min inhibited spore swelling and germination. Additional treatment (5 min) with 5 μM of the reducing agent 2-β-mercaptoethanol (2ME) fully reversed the inhibition effect. Pictures were taken 15 min and 3 h, in the upper and lower rows, respectively, after incubation in water or sweet potato active fraction (SPAF). Bar = 100 μm.

For the spore-germination experiments, the solutions were acidified with 0.1 M HCl, implicating that pH, rather than the organic or amino acids, induced swelling of *R*. *delemar* spores. The fact that the spores swell in acidified solution led to the hypothesis of regulated water uptake by the spores in an acidic conditions and a potential involvement of aquaporin water channels on top of water influx by osmotic gradient [46]. To determine whether AQPs are involved in water uptake during the early stage of germination, we treated the spores with mercury chloride (HgCl_2_), which is expected to decrease AQP water permeability by binding to cysteine (Cys) residues in the AQP channel [41, 43, 47]. Adding HgCl_2_ to the spore suspension in the presence of SPAF led to inhibition of swelling and germination (Fig 2 and Table 2). Treatment of the spores with 5 μM of the reducing agent 2ME reversed the effect of HgCl_2_ [48, 49] and the spores developed in a pattern similar to controls (Fig 2 and Table 2).

**Table 2:** Effect of HgCl_2_ on swelling of *Rhizopus delemar* spores incubated in sweet potato active fraction (SPAF) and germ-tube emergence. Spores were treated with 40 μM HgCl_2_ to inhibit aquaporin activity. Additional treatment with the reducing agent 2-β-mercaptoethanol (2ME, 5 μM) was used to reverse the HgCl_2_ inhibition effect. The assays were performed at pH 4.7. Values are means ± SE (n = 500)

Treatment	pH	Diameter (μm)	Germ-tube emergence (%)
SPAF	4.7	23.1 ± 1.6 *A	97.2 ± 1.2 A
SPAF + HgCl_2_	4.7	7.8 ± 0.6 B	0 B
SPAF + HgCl_2_ + 2ME	4.7	20.5 ± 0.4 A	98.2 ± 1.4 A

* Within each measured parameter different upper case letters are significantly different from each other (P≤ 0.05).

### AQPs in the *R*. *delemar* genome

**Fig 3:**
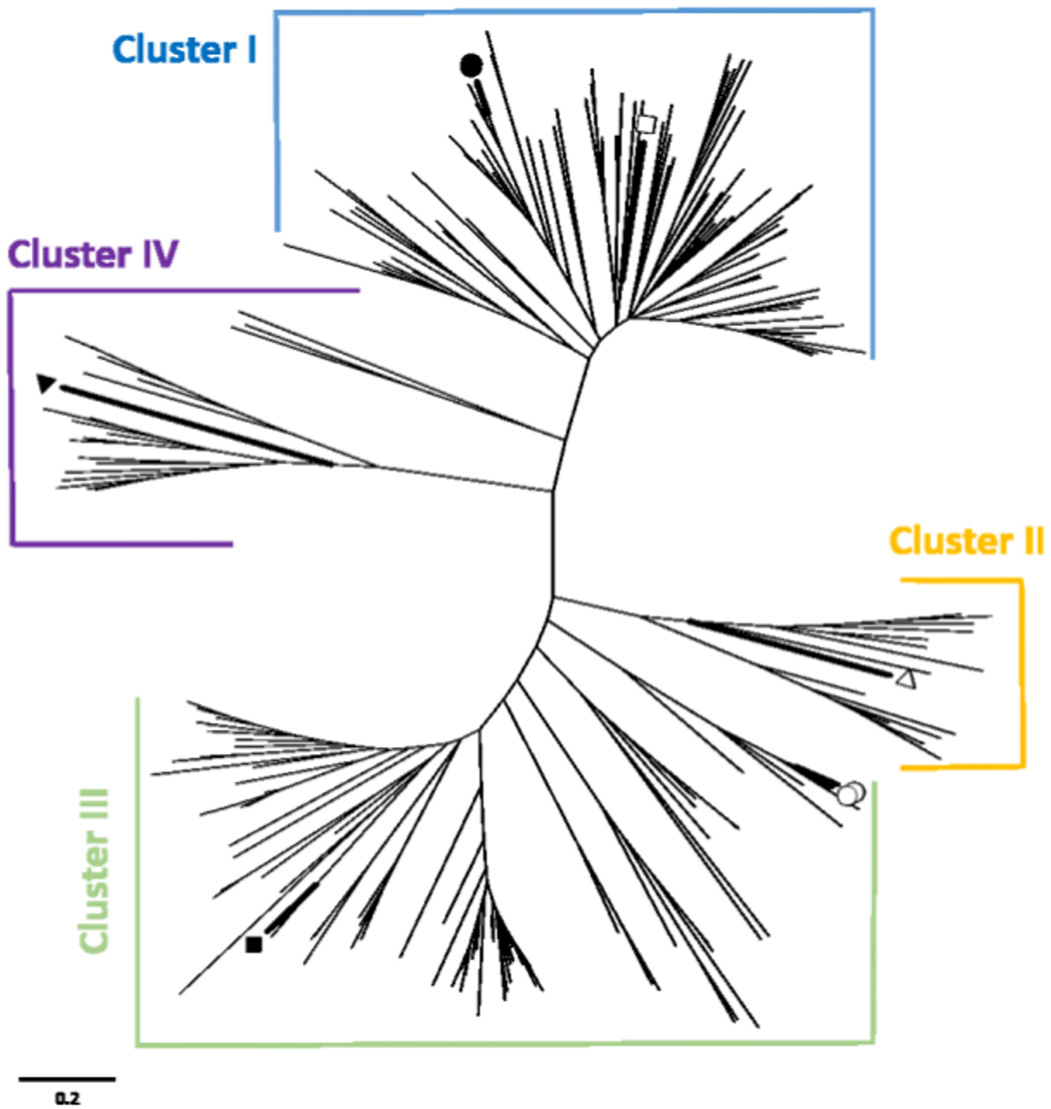
Phylogenetic tree of 231 fungal major intrinsic proteins (MIPs). The MIPs clustered into four distinct groups: Cluster I—putative water channels MIPs (represented by ADC55259|*Saccharomyces cerevisiae* [Black circle] and JF491353|*Terfezia claveryi* [black square]), cluster II—putative aquaglyceroporins MIPs that preferentially transporting small neutral molecules (represented by Lacbi2|671860|*Laccaria bicolor* [open triangle]), cluster III—MIPs that putatively act as water and small neutral molecule transport channels (represented by GAA23030|*S*. *cerevisiae* [black rhombus]), and cluster IV—putative fungal X intrinsic proteins (XIPs) (represented by TmeAQP2|*Tuber melanosporum* [black triangle]). Both *RdAQP1* and *RdAQP2* (open circles) are located in Cluster III, pointing on their potential capability to act as a water channels. Bar represents 0.2 changes.

A search of the *R*. *delemar* genome database [Mucorales Sequencing Project, Broad Institute of Harvard and MIT (http://www.broadinstitute.org)] revealed only two candidate AQP genes, *RdAQP1* and *RdAQP2*, which are common among fungi but are considered to be members of a very small gene family compared to organisms from other kingdoms. Phylogenetic analysis of *R*. *delemar* AQPs using the MEGA 4.0 program (http://www.megasoftware.net/mega4) showed that both RdAQP1 and RdAQP2 are located in Cluster III, of MIPs, that putatively act as water and small neutral molecule transport channels (Fig 3).

### Differential expression of *RdAQP1* and *RdAQP2*

**Fig 4:**
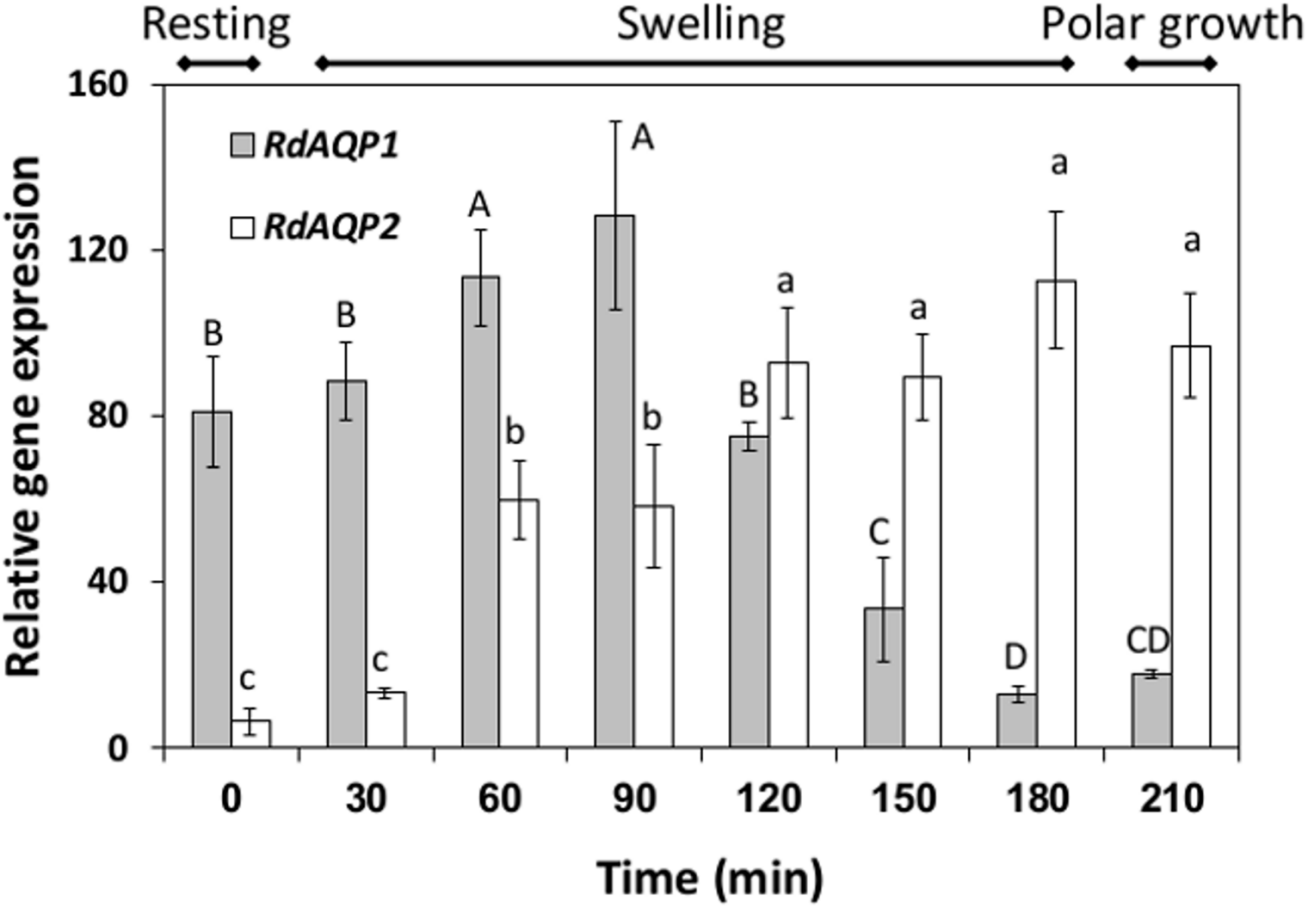
Differential expression of *Rhizopus delemar* putative aquaporin genes (*RdAQP1* and *RdAQP2*) during germination. *R*. *delemar* spores were incubated in SPAF and harvested at three main stages: resting (time 0), swelling (30–180 min) and polar growth (210 min). qPCR was performed on cDNA reverse-transcribed from the RNA extracted from the spores. *Rhizopus 18S* rRNA served as the reference gene. Values of the steady-state levels of gene transcripts were determined as the ratio between two conditions using the 2Δ-ΔCt method [50]. Values are means ± SE. Different upper or lowercase letters above the bars denote significant differences between measurement times in *RdAQP1* or *RdAQP2* respectively (P < 0.05).

The differential expression of the two AQP-encoding genes was monitored in spores from the resting stage, through the swelling stage and to the polar growth stage (Fig 4). Differential expression patterns were revealed for the two genes: *RdAQP1* transcripts were abundant at the resting stage and their expression increased about 1.5-fold during swelling, up to 90 min after induction of germination, followed by a 6.5-fold decrease before and during polar growth (Fig 4). In contrast, *RdAQP2* transcript levels were relatively low at the resting stage and its expression increased steadily from the induction of germination to a peak after 180 min, at the onset of the polar growth stage (Fig 4).

### Predicted topology and localization of RdAQP1 and RdAQP2 proteins

**Fig 5:**
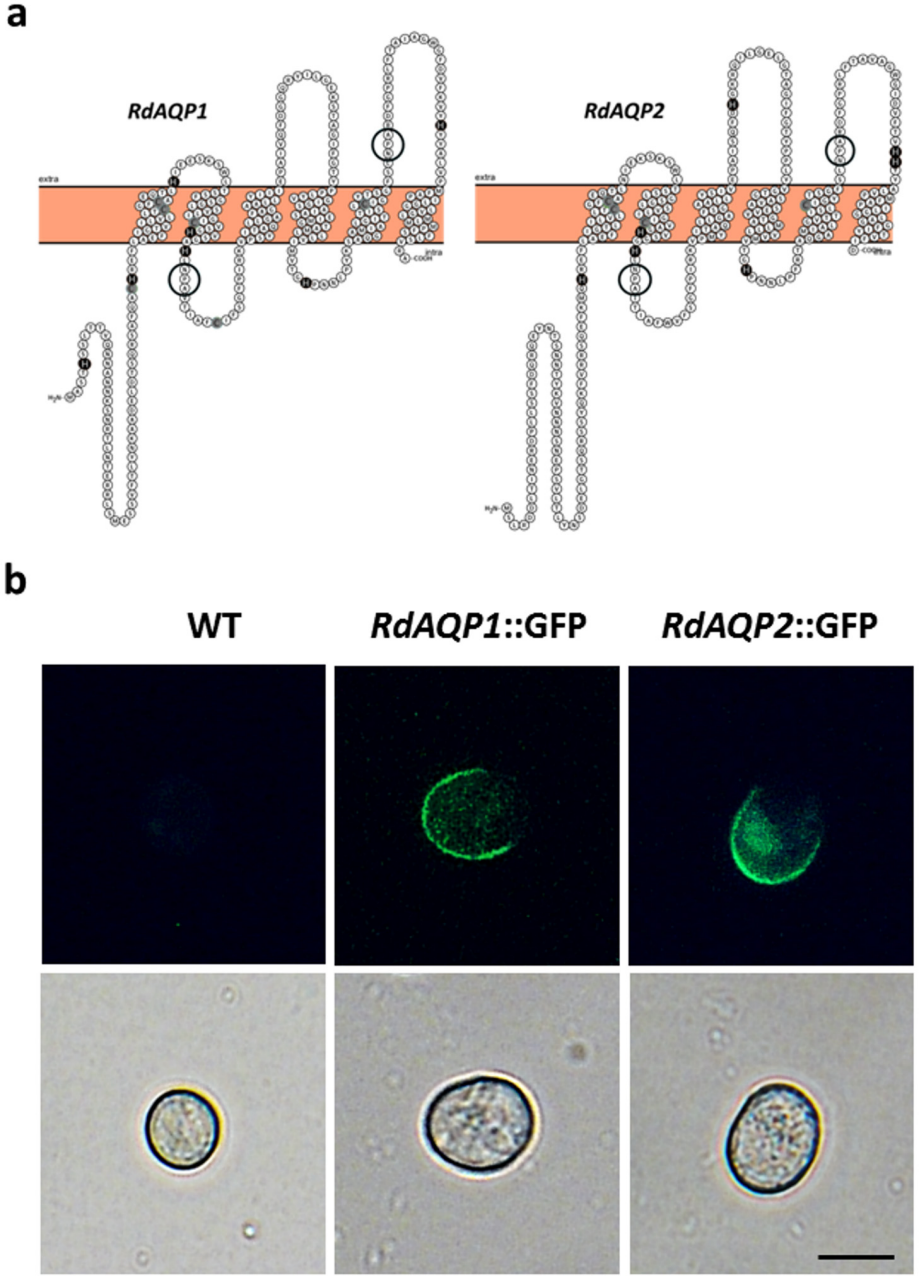
Predicted topology and localization of RdAQP1 and RdAQP2 proteins. (a) Hypothetical prediction of RdAQP1 and RdAQP2 topology by the TMHMM method, drawn by the *Protter-visualize proteoforms* program [53], showing the proteins' six transmembrane domains. The highly conserved NPA domains are circled. The Cys and His amino acids are filled in gray and black color, respectively. (b) Expression of RdAQP1::GFP and RdAQP2::GFP in *R*. *delemar* spores. Bar = 10 μm.

*In-silico* prediction of RdAQP1 and RdAQP2 topology using the TMHMM method (http://www.cbs.dtu.dk/services/TMHMM) confirmed the typical AQP structure of six transmembrane domains (Fig 5a) (reviewed by [51]). For both RdAQP1 and RdAQP2, the predicted protein sequence contained the highly conserved NPA domain which forms the water-specific channel [52]. Fusion of RdAQP1 and RdAQP2 to GFP and transformation into *R*. *delemar* spores suggested that both of the proteins are confined to the intracellular part with higher intensity in the limits of the cell (Fig 5b).

### Functional analysis of RdAQP1 and RdAQP2 in relation to pH conditions

**Fig 6:**
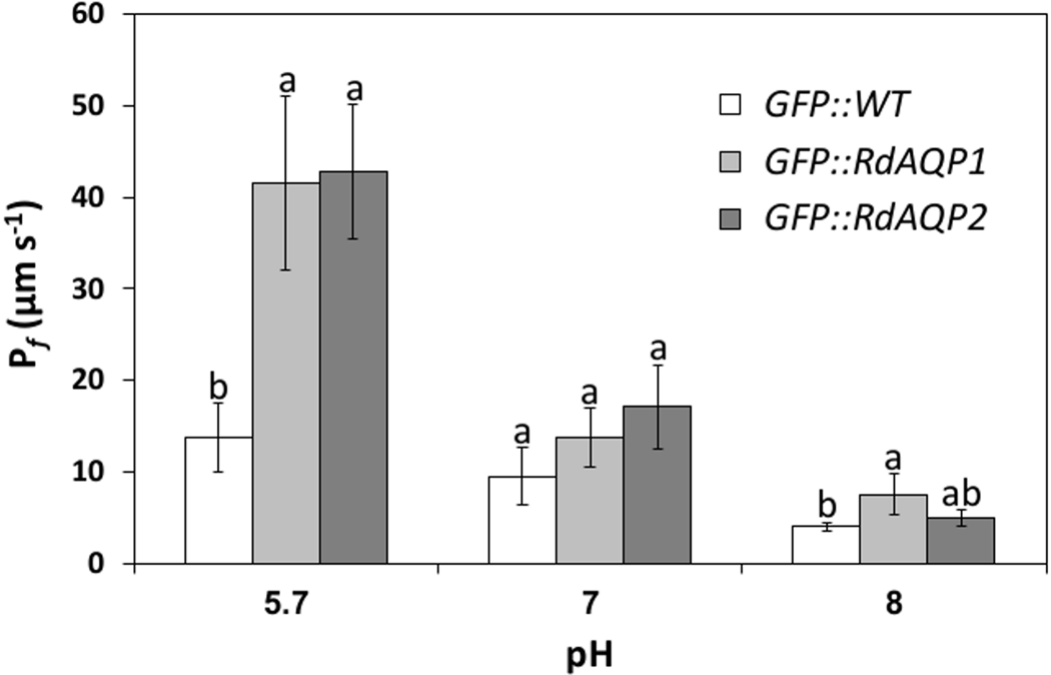
The effect of pH on *Rhizopus delemar* aquaporin (AQP) water permeability. The protoplast osmotic water permeability coefficient (*P*_f_) determined in *Arabidopsis thaliana* protoplasts transiently expressing GFP::AQP (GFP, green fluorescent protein) and in control protoplasts transiently expressing GFP. Values are means ± SE (n = 15). Different lowercase letters above the bars denote significant differences for each pH level in each transformed gene (*P* < 0.05).

AQPs are integral membrane proteins belonging to a larger gene family that functions as water-channel activity or in transport of non-charged molecules such as glycerol, urea, ammonia and CO_2_ [54]. To determine whether the two AQP genes found in the *R*. *delemar* genome function as water channels, each gene was cloned under a constitutive 35S promoter and transiently expressed in the heterologous expression system of *Arabidopsis* protoplasts. Then, the impact of RdAQP1 and RdAQP2 on the osmotic water permeability coefficient (*P*_f_) of the transformed cells was measured. At pH 5.7, the *P*_f_ value of the protoplasts expressing *R*. *delemar* AQPs was significantly higher than for control cells (3.4- and 3-fold for RdAQP1 and RdAQP2, respectively). These results confirmed the role of *R*. *delemar* AQPs as water channels (Fig 6).

As spore swelling was affected by pH, the osmotic water permeability assay was conducted at different pHs (Fig 6). While at pH 5.7, the *P*_f_ value of cells expressing both RdAQP1- and RdAQP2 was about 3-fold higher than in controls, at pH 7.0, the *P*_f_ was similar to that in control cells (Fig 6). A further reduction in *P*_f_ values was observed at pH 8.0. These results demonstrated the critical role of external pH on the water-channel activity of *R*. *delemar* AQPs.

### The role of His residues in pH sensing by RdAQP1

**Fig 7:**
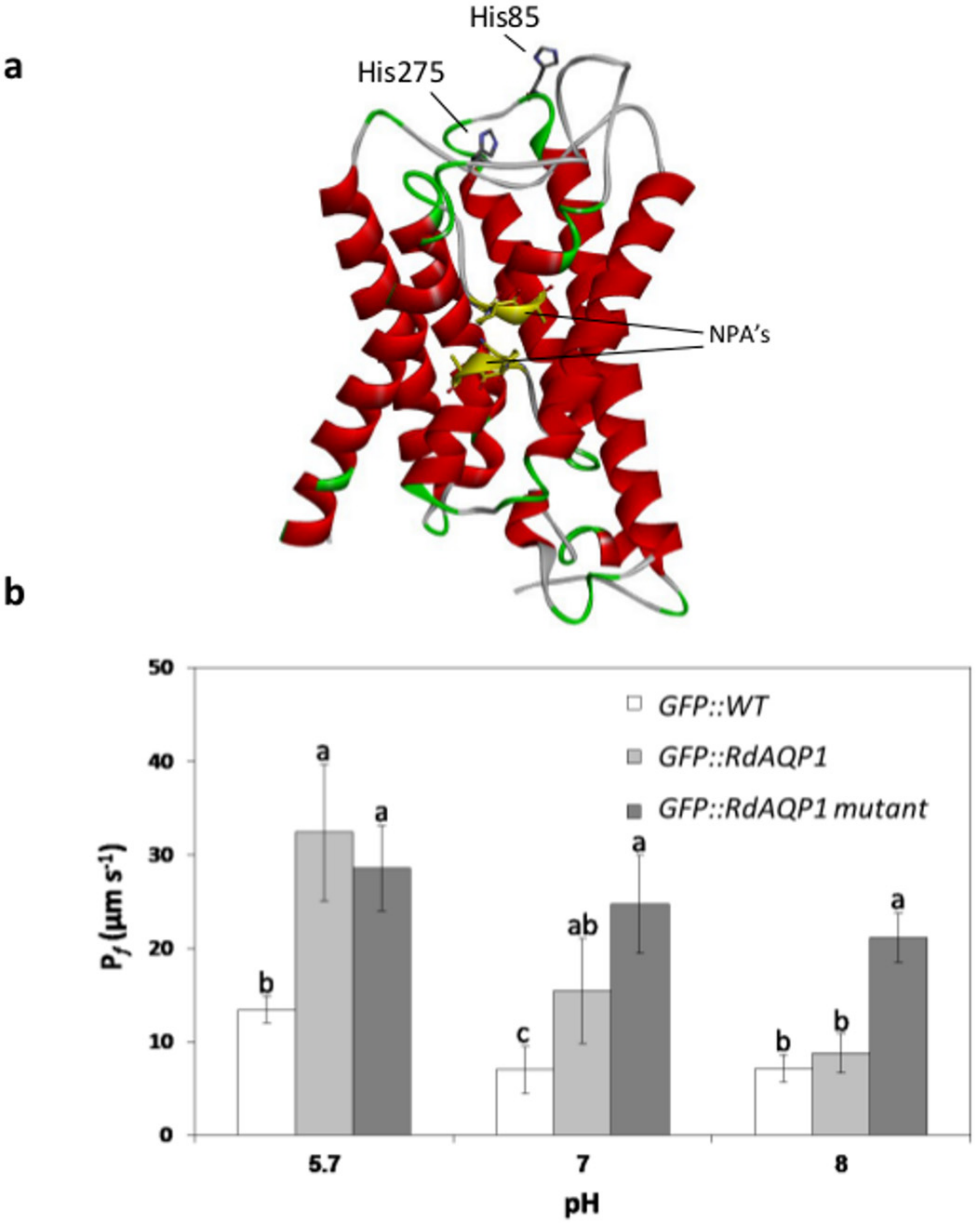
The role of the outer cell His residues in pH sensing. (a) Predicted three-dimensional structure of RdAQP1 showing two His residues (His85 and His275) positioned on the loops of the protein and facing the outside of the cell. (b) *P*_f_ values of GFP::WT (wild type), GFP::RdAQP1 and GFP::RdAQP1 mutant,. Different lowercase letters above the bars denote significant differences for each pH level between fungal types (*P* < 0.05).

Previous studies have proposed a gating mechanism in which the translated AQP protein is regulated by inner or outer cell pH [46, 55]. Those authors suggested that protonation of a conserved His residue will increase or decrease water conductivity of the AQP channel, depending on that residue's position [56]. A hypothetical three-dimensional structure of the RdAQP1protein revealed two His residues at positions 85 and 275, predicted to be located externally and hence be subject to pH-dependent protonation (Fig 7a). A double His mutant (substitution of H85 and H275 with A) was constructed and transformed into *Arabidopsis* protoplasts. The RdAQP1 mutant lost its pH sensitivity as the *P*_f_ values of the transformed protoplasts showed a minor and non-significant decrease when pH was raised from 5.7 to 8.0 (Fig 7b). At pH 8.0, the *P*_f_ value of the RdAQP1 mutant was significantly higher than those of both *RdAQP1*-transformed protoplasts and control protoplasts (Fig 7b). As expected at pH 5.7, the *RdAQP1*-transformed protoplasts differed significantly from the control, while at pH 7.0 and 8.0, no differences were observed between treatments. This experiment suggested that the His residues facing the outside of the cell are involved in RdAQP1 pH sensing.

## Discussion

Spore formation is a very important stage in the fungal life cycle that enables these organisms better preservation under unfavorable conditions [5]. The spore stays in a resting state until it encounters environmental conditions that can support its development. Thus, the spore must be capable of sensing and responding to environmental signals. Spores of different fungal species have diverse requirements for the triggering of germination [5, 8, 9, 14, 15, 57–59]. Nevertheless, spore dehydration during maturation seems to be a fundamental process that is common to all spore-producing fungi. The question of regulation of water uptake during germination is of prime significance for the fungal life cycle [31].

In this study, spore germination of *R*. *delemar* decreased markedly below pH 3.5 and above pH 6.5 (Fig 1), while the optimal pH for germination was between 4 and 5. The time point selected for assessment of the pH effect was 6 h, when the peak germination efficacy was above 90%. As expected, at later time points, the efficacy at the marginal pH values was higher (not shown) suggesting that pH has a modulatory rather than strictly restrictive activity. The effect of pH on spore germination has been documented in previous studies [25, 27]. The intense decrease in germination at low pH compared to the moderate decrease at higher pH implies different mechanisms by which pH affects spore germination. Efficiency of enzyme activities, nutrient availability and proton gradient across the plasma membrane are likely involved in modulation of germination [28, 29]. According to the results of this study, the presence of glucose, at low pH, triggers spore swelling. These results are in accordance with previous studies showing that glucose induces spore swelling in *Rhizopus*, *Fusarium* and *Penicillium* spp. [8, 13, 59–61]. Thanh et al. [59] suggested that glucose and amino acids play important roles in activation and germination of sporangiospores of *Rhizopus oligosporus* and this depends on the uptake potential of specific amino acids and/or glucose [59]. Such mechanism may explain why only glucose induced *R*. *delemar* spore swelling in an acidic environment, while glucose under neutral conditions had reduced effect on the spores (Table 1). Interestingly, acidified sucrose or fructose fractions did not have any effect on spore swelling or germination. Glucose can be replace by fructose or sucrose as a carbon source, but still maximum germination and biomass production required a nitrogen source, phosphate ions and potassium or sodium ions [62, 63]. The specific effect of different sugars on germination has been documented in fungi: while the uptake of glucose is higher in many fungal species, fructose is a better inducer of germination in *B*. *cinerea* ([64] and references therein). Knockout of specific fructose transporter in Botrytis demonstrated delayed fructose-induced germination but both mutants and wild type conidia showed higher affinity to glucose. These results suggest that fructose-induced germination did not depend on transport but possibly on intracellular sensing mechanisms.

*R*. *delemar* spores treated with acidified water (Table 1) did not respond by swelling or germination, and did not aggregate. In addition, a treatment with the AQP blocker HgCl_2_ inhibited spore swelling and germination (Fig 2 and Table 2) pointed to water uptake and hydration of the spore as a crucial step in the very early stages of fungal spore germination. These results are in agreement with a study in which *Fusarium graminearum* and *Fusarium poae* spore hydration was inhibited as a means of preventing spore germination [65].

To better understand the relation between water uptake and pH conditions and the mechanism that enables water uptake by the spore at an early stage of germination, we analyzed the *R*. *delemar* genome to identify genes that could potentially act as water channels. The presence of only two candidate AQPs in the *R*. *delemar* genome corresponded with other fungi which present a relatively small number of these genes compared to mammals and plants [66]. Phylogenetic analysis clustered these proteins with a group of fungal major intrinsic proteins (MIPs) suggested to act as water channels (Fig 3). Taking into account the important activity of AQPs in the transitioning of small and uncharged molecules such as water, acids, carbon dioxide, glycerol and ammonia, the small number of these genes in *R*. *delemar* and other fungal genomes suggests a non-selective role for these proteins [67–70]. The protein structure of AQPs is conserved and can be found in organisms from all kingdoms [69, 71–74]. According to the transmembrane domains and the expected role in water uptake, fusion of the two genes to GFP and transformation into *R*. *delemar* spores localized the two encoded proteins to the cell membrane (Fig 5b). The proteins' similarity of *R*. *delemar* AQPs to the plasma membrane-intrinsic protein (PIP; Fig 3), AQP subfamily in other organisms [75, 76], is another indication for localization on the outer membrane.

Expression analysis of *RdAQP1* and *RdAQP2* revealed opposite patterns for the two genes as germination progressed (Fig 5). While *RdAQP1* transcripts were abundant in the spore resting stage and during the early stages of germination, *RdAQP2* transcripts were low in the resting stage and increased as germination progressed (Fig 5). These results suggest that *RdAQP1* is spore-specific and therefore it may have a primary role in spore hydration. *AQY1*, the *Saccharomyces cerevisiae* spore-specific AQP, functions during the sporulation stage, facilitating spore dehydration [77]. In addition, overexpression of *AQY1* mediates water efflux and increases freeze tolerance of yeast cells. Another interesting mycorrhizal interaction has been reported for *Phaseolus vulgaris*, suggesting that the symbiont is involved in the transfer of water to the host, thereby improving its tolerance to drought stress [78, 79], or improving urea, glycerol and/or NH_3_ uptake [80, 81]. The role of AQPs in yeast and filamentous fungi has been reviewed [66, 82] with respect to three main aspects: sporulation, freeze tolerance and mycorrhizal associations [77, 80, 82–85]. Since AQPs might act as a transfer channel for small and non-charged molecules rather than water channels, it was important to perform a biological assay that would elucidate their role (Fig 6). *RdAQP1* and *RdAQP2* were expressed in *Arabidopsis* protoplasts which were then exposed to hypotonic solution to calculate plasma membrane *P*_f_ potential [39]. Although the *P*_f_ values of the plasma membrane, when *RdAQP1* and *RdAQP2* expressed, is lower than those found in other fungal AQPs [80], they were found to be significantly higher than controls and probably contributed to water uptake of *R*. *delemar* spores during the early stages of spore germination. In contrast to an earlier work that did not find any correlation between environmental osmotic pressure and water-uptake capacity [13], the current work demonstrated rapid water uptake by the transformed protoplasts as the osmotic pressure of the solutions changed from isotonic (600 mOsm) to hypotonic (500 mOsm). To the best of our knowledge, this is the first evidence for regulated water uptake in fungi during the early stages of spore germination.

In this work, we found that *R*. *delemar*, like several other pathogenic fungi, requires a specific pH range for optimal germination (Fig 1) [25, 27]. In addition, water uptake occurred at the early stages of germination and inhibition of water uptake using HgCl_2_ caused inhibition of spore swelling and germination (Fig 2). Both of RdAQP1 and RdAQP2 present Cys residues located near to the conserved NPA motifs (C240 and C256, respectively). Due to their proximity to these motifs and according to their trans-membranal predicted localization they are assumed to be targets for HgCl_2_ binding.

To understand whether water uptake by *RdAQP1* and *RdAQP2* is regulated by ambient pH, water-uptake experiments were carried out under several pH conditions (Fig 6). Reduction in *RdAQP1* and *RdAQP2 P*_f_ levels as the ambient pH increased suggests pH regulation of *R*. *delemar* water channels and might provide a practical explanation for the specific range of ambient pH required for optimal spore germination. pH regulation of AQPs has been vastly documented in the literature [46, 55, 56, 86–88].

Different regulation mechanisms that directly affect the spatial structure of AQPs have been proposed (reviewed by [89]): (i) phosphorylation of S residues within the N or C termini; (ii) oligomeric state of the protein (heteromerization); (iii) gating by high solute concentration and pressure pulses, and (iv) regulation by pH. With respect to pH regulation, several works have shown that His residues are protonated in acidic environments and contribute to the "gating mechanism" of the water channel [46, 87]. Protonation on a conserved His residue of AQPs could either lead to activation or inhibition of the water channels activity. Most of plant's aquaporins of the PIP subgroup are inhibited by cytosol acidosis [46, 55] while external acidic pH or low Ca2+ increased the water permeability in bovine's AQP0 [56]. Depending on the position of the His residue (on the loop, inside or outside the cell), the protonation will switch the water channel's permeability on or off [87]. It seems that the flexibility of pH regulation through the His residue position enables an efficient mechanism of adaptation to environmental conditions. To better understand the mechanism by which pH regulates RdAQP1 function, two His residues positioned on the outer cell loops (Fig 7a) were replaced with A. In agreement with earlier studies, the *RdAQP1* mutant lost its pH sensitivity and its *P*_f_ became unaffected by pH (Fig 7b), suggesting that one (or both) of the His play a role in pH sensing [46].

In conclusion, this study suggests a critical role for extracellular pH in *R*. *delemar* germination by altering fungal AQP function and making it a potential pH sensor. As wounded vegetables and fruit present acidic pH, it makes sense that the optimal pH range for germination is adapted to the relevant host pH. This AQP-mediated adaptation enables the spores to hydrate under favorable conditions and to commence germination.
